# Learning-Related Neuronal Activation in the Zebra Finch Song System Nucleus HVC in Response to the Bird’s Own Song

**DOI:** 10.1371/journal.pone.0041556

**Published:** 2012-07-25

**Authors:** Johan J. Bolhuis, Sharon M. H. Gobes, Nienke J. Terpstra, Ardie M. den Boer-Visser, Matthijs A. Zandbergen

**Affiliations:** 1 Departments of Psychology and Biology, Cognitive Neurobiology and Helmholtz Institute, Utrecht University, Utrecht, The Netherlands; 2 Neuroscience Program, Wellesley College, Wellesley, Massachusetts, United States of America; 3 Behavioural Biology, Institute of Biology, Leiden University, Leiden, The Netherlands; Rutgers University, United States of America

## Abstract

Like many other songbird species, male zebra finches learn their song from a tutor early in life. Song learning in birds has strong parallels with speech acquisition in human infants at both the behavioral and neural levels. Forebrain nuclei in the ‘song system’ are important for the sensorimotor acquisition and production of song, while caudomedial pallial brain regions outside the song system are thought to contain the neural substrate of tutor song memory. Here, we exposed three groups of adult zebra finch males to either tutor song, to their own song, or to novel conspecific song. Expression of the immediate early gene protein product Zenk was measured in the song system nuclei HVC, robust nucleus of the arcopallium (RA) and Area X. There were no significant differences in overall Zenk expression between the three groups. However, Zenk expression in the HVC was significantly positively correlated with the strength of song learning only in the group that was exposed to the bird’s own song, not in the other two groups. These results suggest that the song system nucleus HVC may contain a neural representation of a memory of the bird’s own song. Such a representation may be formed during juvenile song learning and guide the bird’s vocal output.

## Introduction

Birdsong learning is a prominent model system for the study of the neural mechanisms of learning and memory [1], [2], [3], [4], [5], [6], [7]. In addition, there are behavioral similarities between the way that songbirds learn to sing their songs and human infants acquire speech and language, that are absent in non-human primates who do not seem to have a capacity for vocal imitation [8], [9], [10]. Songbirds such as zebra finches (*Taeniopygia guttata*) learn their songs from an adult conspecific, or ‘tutor’, during a sensitive period early in life [11], [12]. In the zebra finch, song learning occurs in two partially overlapping phases: a memorization phase during which the bird forms an auditory memory of the tutor song, and a sensorimotor phase when the animal starts vocalizing and eventually develops a ‘crystallized’ song that may resemble that of its tutor. In both human infants and juvenile songbirds there is a sensitive period for learning early in life, and a transitional phase called ‘babbling’ and ‘subsong’, respectively.

**Figure 1:**
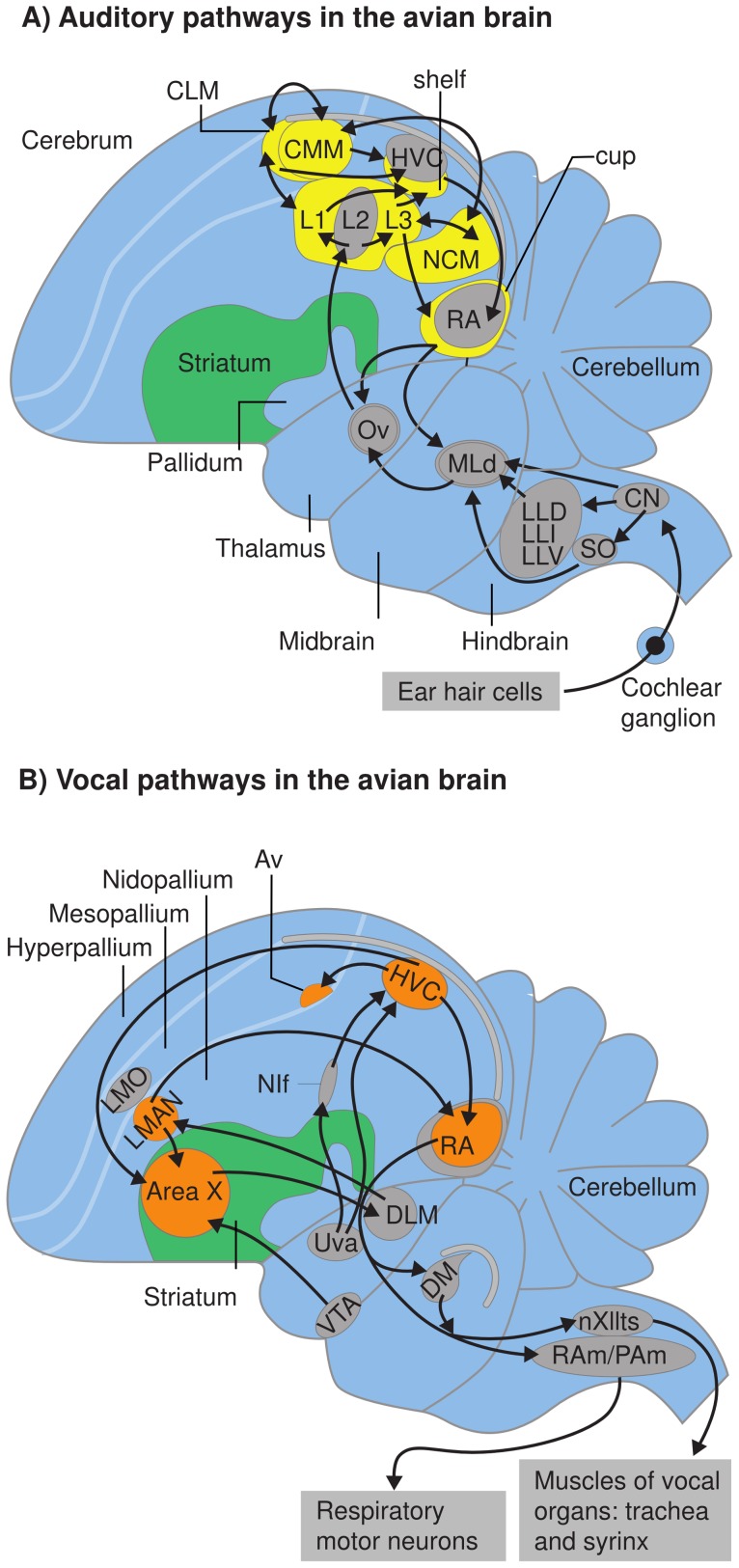
Schematic diagrams of composite views of parasagittal sections of the songbird brain. A. Diagram of a songbird brain giving approximate positions of nuclei and brain regions involved in auditory perception and memory. Yellow areas represent brain regions that show increased neuronal activation when the bird hears song. B. Diagram of a songbird brain giving approximate positions of nuclei and brain regions involved in vocal production and sensorimotor learning. Orange nuclei in the song system show increased neuronal activation when the bird is singing. Abbreviations: CLM, caudal lateral mesopallium; CMM, caudal medial mesopallium; DLM, nucleus dorsolateralis anterior, pars medialis; HVC, acronym used as a proper name; L1, L2, L3, subdivisions of Field L; LMAN, lateral magnocellular nucleus of the anterior nidopallium; NCM, caudal medial nidopallium; nXIIts, tracheosyringeal portion of the nucleus hypoglossus; RA, robust nucleus of the arcopallium. Modified and reproduced, with permission, from references [9] and [60], copyright 2010 Nature Publishing Group. All rights reserved.

The behavioral similarities between song and speech are matched by neural parallels [8], [9]. In humans and songbirds there is a similar neural dissociation between brain regions involved in vocal production and sensorimotor learning on the one hand, and those involved in auditory perception and memory on the other 4,5,9,13,14. In humans, speech and language functions are subserved by a neural network connecting brain regions in the frontal and temporal lobes. Roughly, speech production mainly involves Broca’s area and associated regions in the frontal lobe, while speech perception and memory is subserved mainly by regions in the temporal lobe, including Wernicke’s area [8], [9]. There are neural analogies and homologies between the brains of birds and mammals, that have led to a complete overhaul of the nomenclature of the avian brain [15], [16]. In songbirds, song production and sensorimotor learning involves a network of interconnected brain nuclei known as the song system (Fig. 1B) [2], [6], [9], [17]. The song system is comprised of two major pathways. First, the song motor pathway (SMP) is involved in song production and certain aspects of song learning [6]. The SMP is a posterior motor pathway connecting the HVC (acronym used as a proper name), the robust nucleus of the arcopallium (RA) and the tracheosyringeal portion of the nucleus hypoglossus (nXIIts). Second, the anterior forebrain pathway (AFP) is essential for sensorimotor learning and adult song plasticity [1]. The AFP is an anterior cortical–basal ganglia–thalamic loop that originates in HVC and passes through Area X (part of the avian basal ganglia [18]), the thalamic nucleus dorsolateralis anterior, pars medialis (DLM) and the lateral magnocellular nucleus of the anterior nidopallium (LMAN), and eventually connects with the motor pathway at the nucleus RA. Brain regions outside the song system that are involved in perception and recognition of song include the caudomedial nidopallium (NCM) and the caudomedial mesopallium (CMM) [4], [5], [19], [20], [21], [22], [23], [24], [25], [26], [27], [28], [29] (Fig. 1A).

Evidence for the involvement of the NCM and the CMM in song perception was provided by studies showing that exposure of zebra finches or canaries (*Serinus canaria*) to conspecific song did not lead to increased neuronal activation in nuclei in the song system, but in these two forebrain regions, among others [19], [20]. First support for the NCM as (part of) the neural substrate for auditory memory came from studies investigating habituation-like processes. Repeated exposure to a song leads to decreased immediate early gene (IEG) expression [21] and to decreased electrophysiological responding of units in the NCM to that song [22]. On the basis of their results, Chew et al. [22] suggested that “the NCM is specialized for remembering the calls and songs of many individual conspecifics”. Subsequent research by Bolhuis and colleagues showed that the NCM is the likely site of the neural substrate of tutor song memory [4], [5]. These authors found a significant positive correlation between the strength of song learning (the number of elements that males had copied from the song of their tutor) and neuronal activation (measured as the expression of IEGs) in the NCM [25], [26], [27]. The relationship between neural activity in the NCM and the strength of song learning was confirmed in an electrophysiological study [28]. The hypothesis that the NCM contains the neural substrate for a representation of tutor song memory received further support in a study involving adult zebra finch males that received discrete neurotoxic lesions to this structure. NCM lesions significantly impaired tutor song recognition, while song production was left intact [13]. More recently, London and Clayton [29] demonstrated that a molecular response (regulated by extracellular signal regulated kinase, ERK) in the NCM is necessary for normal song learning to occur in juvenile zebra finches. In addition, in juvenile zebra finch males in the middle of the sensorimotor phase, IEG expression was greater after exposure to tutor song than after exposure to novel song, suggesting that the NCM is involved in tutor song recognition memory in juveniles [30].

In the song system, neuronal activation (measured as the expression of IEGs) is significantly increased during singing [23]. Electrophysiological research in zebra finches has shown that neurons in nuclei of the song system (in particular, lMAN, Area X, HVC and RA; see Fig. 1B) are responsive to song, particularly to conspecific song [31]. In adult males, most neurons in these nuclei respond more to the bird’s own song (BOS) than to the tutor song [31], [32], or to the song of an unfamiliar conspecific [33].

On the basis of all these different studies, Bolhuis and colleagues suggested that the NCM may be (part of) the neural substrate for the representation of the tutor song, while the song system may contain a neural representation of the bird’s own song [4], [5], [9]. If this hypothesis is correct, it is predicted that there will be neuronal activation (measured as the expression of IEGs) in the song system related to BOS. Previously we found a significant positive correlation between the strength of song learning and neuronal activation (measured as IEG expression) in the NCM of adult zebra finches that were re-exposed to the tutor song, not in birds that were exposed to a novel conspecific song or to BOS [27]. Here we investigated whether there is BOS-induced IEG expression in the song system in the same subjects. We found that similarity with the tutor song (a measure of the strength of song learning) correlated significantly with IEG expression in the song system nucleus HVC in males that were exposed to BOS, and not in males that were exposed to tutor song or to novel song. These findings are consistent with a role for HVC in the neural representation of the bird’s own song.

## Results

Socially reared adult zebra finch males were exposed to either the song of their tutor, to their own song (BOS) or to the song of an unfamiliar conspecific, and the expression of the immediate early gene (IEG)_product Zenk (a marker for neuronal activation) was measured in the song system nuclei HVC, RA and Area X. We determined the relationship between Zenk expression and the similarity of the subject’s song with that of their tutor, as a measure of the strength of song learning.

### Song Similarity

The mean percentage of elements that the experimental birds copied from the tutor song (song similarity) was 49.4% (±4.4, SEM), while the mean percentage of elements shared between the songs of unrelated males was 10.0% (±2.2, SEM). The mean number of elements that the experimental males copied from the tutor did not differ significantly between experimental groups, nor did the mean length of recorded stimulus songs or the number of different elements in these songs.

### Preference Tests

Preference scores could not be calculated for all birds because some did not learn to press both keys (n = 4) or one key (n = 3), or because of technical problems (n = 1), thus leaving a total of 21 birds with preference scores (n = 7 in the TUTOR group, n = 8 in the BOS group and n = 6 in the NOVEL group). The mean preference for the tutor song was significantly greater than 50% (chance level). For all experimental birds: 64% ±2.6 (mean ± SEM), p<0.001; for birds in the TUTOR group: 66% ±3.8, p<0.01; for birds in the BOS group: 61% ±4.3, p<0.05 and for birds in the NOVEL group: 66±5.7, p<0.05. There was no significant correlation between arcsine transformed preference scores and song similarity.

### Zenk Expression in Different Brain Regions

**Figure 2:**
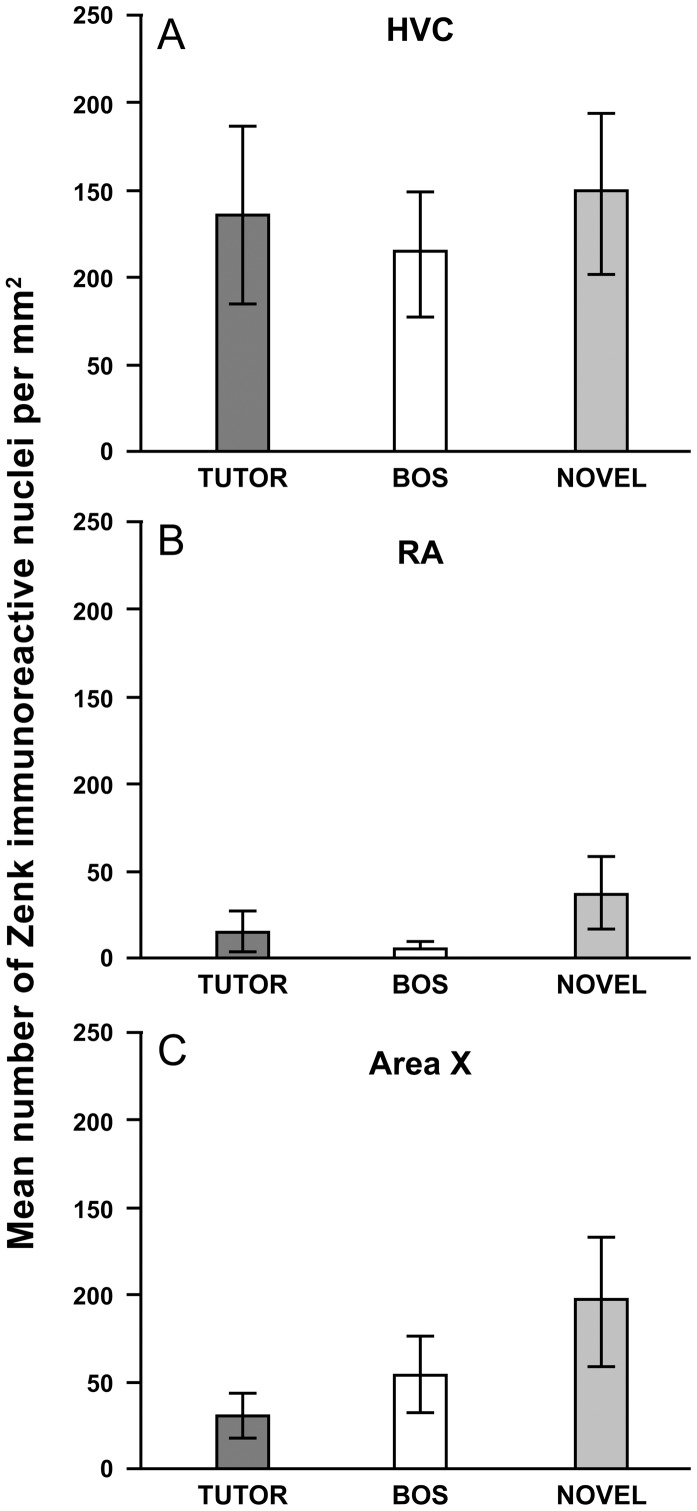
Mean (± SEM) number of Zenk-positive nuclei per mm^2^ in the song system brain nuclei (A) HVC, (B) RA and (C) Area X, for groups of male zebra finches exposed to tutor song (TUTOR), bird’s own song (BOS) or novel conspecific song (NOVEL).

Figure 2 shows the mean number of Zenk immunopositive cells in the three brain regions (HVC, RA and Area X) for the three experimental groups. The overall repeated-measures ANOVA revealed significant effects of the factor Brain Region (*F*(2,48) = 35.1; *p*<0.0001). There was no significant effect of Group (*F*(2,24) = 0.9), and no significant interaction between Brain Region and Group (*F*(4,48) = 0.6). Separate one-way ANOVAs for the HVC, RA and Area X also revealed no significant effects of Group for any of these brain regions.

### Correlations Between Percentage of Song Elements Copied and Zenk Expression

**Figure 3:**
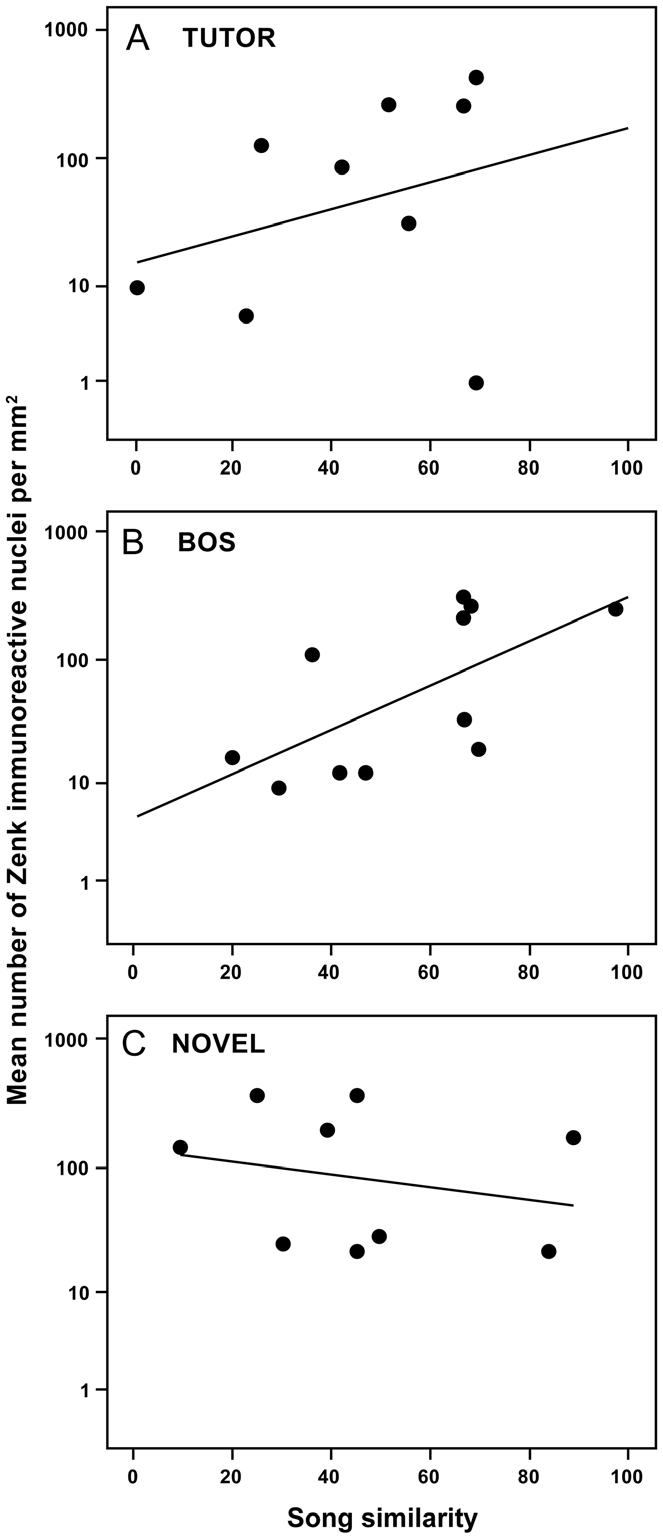
Scatter plots of mean number of Zenk immunoreactive cell nuclei per mm^2^ in the song system nucleus HVC, in relation to the percentage of song elements copied from the tutor song (song similarity) for groups of birds exposed to (A) tutor song (TUTOR), (B) bird’s own song (BOS) or (C) novel conspecific song (NOVEL). Note that y-axis has a log scale.

The correlation coefficients for the relationship between the percentage of song elements copied from the tutor song and the number of Zenk positive neurons in the HCV, RA and Area X are shown in Table 1. Fig. 3 shows scatter plots of the number of Zenk-immunoresponsive nuclei and similarity between songs of experimental males and their tutors in the HVC for groups TUTOR, BOS, and NOVEL. There was a significant positive correlation between the number of Zenk-immunoreactive nuclei and the number of elements copied, only in the HVC in group BOS. There were no significant correlations between the number of Zenk immunoreactive nuclei and number of elements copied in any of the other groups in any of the sampled regions. Visual inspection of the scatter plot for the TUTOR group in Fig. 3A suggests that the lack of a significant correlation may be due to one individual ‘outlier’ (bottom right in Fig. 3A). When this subject was excluded, regression analysis revealed a significant correlation between IEG expression (log transformed) and song similarity: r = .76, p<0.05. However, there is no formal reason (i.e. true statistical outlier or issues with the experimental procedure) to exclude this subject from the analysis. IEG expression in the NCM of the same subject in our previous study [27] was within the range of values, and not particularly low.

**Table 1:** Correlation coefficients of the relationship between the number of elements copied from the tutor song and Zenk expression in the HVC, RA and Area X for groups of birds exposed to the tutor song (TUTOR), bird’s own song (BOS), or novel conspecific song (NOVEL).

Stimulus	TUTOR		BOS		NOVEL	
	r	p-value	r	p-value	r	p-value
HVC	0.30	NS^a^	0.65	0.029	−0.24	NS
RA	0.43	NS	0.40	NS	−0.53	NS
Area X	0.14	NS	0.52	NS	−0.373	NS

a NS, Not Significant; p-values <0.05 were considered significant.

## Discussion

There were no significant differences in overall neuronal activation between adult zebra finches exposed to tutor song, bird’s own song (BOS) or novel conspecific song in any of the song system nuclei (HVC, RA and Area X) that we sampled. However, we found a significant positive correlation between the strength of song learning and IEG expression in the song system nucleus HVC in males that were exposed to their own song (BOS), and not in males exposed to either tutor song or to a novel conspecific song. Previously, using the same subjects, we found song learning-related neuronal activation in the NCM in males exposed to tutor song, not in males exposed to BOS or novel song [27]. Taken together, these findings are consistent with the suggestion [4], [5], [9] that the NCM contains (part of) the neural representation of the tutor song, while the song system nucleus HVC may be a locus for a neural representation of the bird’s own song (BOS). The latter hypothesis is supported by an extensive body of electrophysiological evidence showing that neurons in the song system (including HVC) are preferentially responsive to BOS [31], [32], [33], to which the present study adds evidence for learning-related IEG expression in response to BOS. In addition, in both electrophysiological [31], [33] and IEG studies [23] it has been found that neurons in HVC (as well as in the song system nuclei LMAN, Area X and RA) are activated when the bird sings. The memory of the BOS may function as a ‘motor program’ that guides the animal’s vocalizations [4], [34].

The overall level of IEG expression in response to song in the song system nuclei in the present study was considerably lower than in the NCM and the CMM of the same subjects found in our previous study [27]. A similar differential responsiveness was found in previous studies involving immunocytochemical analysis of the IEG protein products Zenk and Fos [25] and in situ hybridization of the IEG *ZENK* [23], in birds that were exposed to song (including BOS) while not singing themselves. Mello and Jarvis [35] discussed the apparent paradox of robust electrophysiological responsiveness of song system neurons to song, particularly BOS, and the very low levels of IEG expression in the song system to these same stimuli. The authors suggested that the paradox could be resolved because most of the early electrophysiological analyses showing significant responding in the song system were conducted in anaesthetized birds, while neuronal activity was thought to be greatly reduced in awake birds [35], [36]. However, several studies in awake swamp sparrows (*Melospiza georgiana*) have demonstrated significant electrophysiological responsiveness in song system nuclei, including the HVC [37], [38], [39]. Prather and Mooney [36] suggested that species differences in responsiveness to BOS in the HVC are due to differences in song repertoire size, which is limited to one song in the zebra finch, while swamp sparrows have several songs in their repertoire. However, electrophysiological recordings in the HVC of juvenile zebra finches has revealed modest but significant responsiveness to BOS [40]. The present findings suggest that although BOS does not induce a significant overall increase in IEG expression in the zebra finch HVC, there is a significant correlation between neuronal activation and the strength of song learning in this brain region when the birds are exposed to BOS.

Although we did not find a significant correlation between song similarity and IEG expression in response to BOS in Area X or RA, we cannot exclude the possibility that these nuclei are involved in a neural representation of the BOS. As we have seen, electrophysiological responsiveness and IEG expression patterns do not always coincide, and several electrophysiological studies have reported preferential responding to BOS in the song system nuclei Area X, lMAN and RA in addition to HVC [31], [32], [33], [41]. In an electrophysiological study of zebra finches with denervated vocal organs (that often had impaired vocal learning), Solis and Doupe [41] found that numerous neurons in Area X showed preferential responding to both BOS and tutor song. The authors suggested that responsiveness to both these stimuli is important for shaping responses in the anterior forebrain, including Area X.

The present results do not rule out the possibility of a neural representation of a memory of the tutor song in HVC. There was a non-significant trend towards a correlation between song learning strength and Zenk expression in HVC in response to tutor song (Fig. 3), that became significant when one subject was excluded from the analysis. On average, there was considerable overlap between BOS and the song of the tutor. Thus, the greater the song similarity score in Fig. 3, the more the tutor song will resemble the bird’s own song. This relationship may explain the non-significant trend in the TUTOR group, which may reflect activation of a representation of BOS rather than of the tutor song. Alternatively, the significant correlation observed in the BOS group might reflect the activation of a representation of the tutor song. Bolhuis and Gahr [4] argued for a role for the NCM in the representation of tutor song memory, but they also entertained the possibility that the NCM may be a ‘relay station’ and that this neural representation is stored elsewhere. Other authors have argued that the song system contains a neural representation of tutor song memory [42], [43], [44], [45], [46], [47] (see [4] for a detailed discussion). Alternatively, it may be that there is a neural representation of the memory of the tutor song in both the NCM and in HVC or another song system nucleus [14]. If that is the case, the question arises which of these representations functions as a ‘template’ [4], [11], i.e. a neural representation of the tutor song that is necessary for vocal learning [14]. If the hypothesis that such a representation is located in the NCM is correct, we predicted [13], [25] that lesions to the NCM of juvenile zebra finch males should impair not only the formation of tutor song recognition memory but also song development. So far, this hypothesis has been tested only indirectly, in a study that investigated whether IEG expression in the NCM is necessary for song learning [29]. London and Clayton [29] infused an inhibitor of the activation of extracellular signal regulated kinase (ERK) in the NCM of juvenile zebra finches during tutoring sessions in which they were exposed to a song tutor. ERK regulates the transcription of *ZENK*, as well as other immediate early genes such as *c-fos* and *Arc* [48]. Juveniles that received this treatment during tutoring sessions, developed poor imitations of the tutor song while normal discrimination of songs was unaffected. Birds that received an inactive compound that is structurally similar to the inhibitor did develop songs that resembled the tutor song. These findings are consistent with the NCM containing a neural representation of the tutor song that is important for vocal learning, but they do not exclude the possibility that simultaneous activity in the song system is equally important for song acquisition [4], [14], [25], [49].

The present results support the suggestion of a neural dissociation between brain regions involved in vocal production and sensorimotor learning on the one hand, and auditory perception and memory on the other [4], [5], [9], [13]. Such a dissociation has a parallel in human speech and language, with Broca’s area mainly involved in speech production and Wernicke’s area mainly subserving speech perception and memory [9]. In both humans and songbirds, these two systems do not operate in isolation, but are thought to interact continually [4], [9]. Such interaction can be seen early in development in both human infants and juvenile songbirds (see [9] for a detailed discussion).

In songbirds, evidence from a number of studies suggests that a representation of BOS in the song system emerges gradually. The white-crowned sparrow (*Zonotrichia leucophrys*) is a songbird in which there is no overlap between the memorization and sensorimotor phases. An electrophysiological study involving this species revealed that neurons in the HVC of juvenile males in the memorization phase showed no preference for songs with which they were tutored (compared to other songs). Later, in the sensorimotor phase when the males started to sing themselves, HVC neurons showed preferential responding to BOS – even when compared to tutor song [50]. More recently, an electrophysiological study involving developing zebra finches, in which memorization and sensorimotor phases overlap, suggests that recording sites in the HVC show transient preferential responding to the song of their tutor [40]. In males in their early sensorimotor period (35–69 days after hatching), responsiveness of HVC recording sites was greater to tutor song than to BOS, novel song, heterospecific song or white noise; in some cases the preference was significant. The level of responsiveness of HVC recording sites did not correlate with the similarity of BOS to the tutor song. Later in development (more than 70 days after hatching) HVC recording sites that had preferentially responded to the tutor song switched to a preference for BOS. Taken together, these findings suggest that the HVC is important for song production in the plastic song phase and that during this phase neurons in HVC acquire their preferential responsiveness to the BOS.

Nick and Konishi’s results in juvenile zebra finches [40] allow for the possibility of a (transient) representation of tutor song memory in the HVC, that may act as a ‘template’ for vocal learning. Alternatively, these findings may not reflect a true ‘template’ in HVC, but could be a manifestation of transient constraints on auditory input to the song system. Evidence consistent with either of these developmental scenarios was obtained in a recent study involving permanent and transient lesions to the song system nucleus NIf [49], the primary brain structure through which auditory signals reach HVC [51], [52] (Fig. 1B). Juvenile zebra finches received bilateral lesions to NIf, prior to first exposure to their tutor, or NIf was transiently inactivated during tutor sessions [49]. In both cases NIf inactivation led to significantly impaired song learning, measured as the similarity of the bird’s own song to its tutor’s song [49]. These findings suggest that auditory input to HVC is important at the onset of song tutoring in juveniles. It remains to be investigated whether during first exposure to the tutor, a representation of BOS is formed in HVC directly, or via a transient representation of (or input biased to) the tutor song, as suggested by the electrophysiological findings of Nick and Konishi [40]. In addition, it may be that a representation of the tutor song in the NCM [4], [9] is also a necessary prerequisite for vocal learning [29], possibly through an interaction between the NCM and HVC at the juvenile stage [4], [9], [14].

## Materials and Methods

### Subjects

We used 29 male zebra finches (*Taeniopygia guttata*), reared socially in the breeding colony of the department of Behavioural Biology, Leiden University. Birds were reared and maintained as described in a behavioral study in which 15 of them participated [53]. Briefly, birds received water and food ad libitum and were maintained on a 13.5/10.5 h light/dark cycle. The males remained with both parents and their siblings until 74±12 days (mean ±SD) after hatching. Thus, during the sensitive period for song learning the experimental birds stayed with their father, who acted as their song tutor. From then onwards, they were housed in same sex groups except during preference tests at 112±11 (mean ±SD) days after hatching and when their songs were recorded.

### Preference Tests

The preference testing procedure has been described elsewhere [53], [54], [55]. Briefly, operant conditioning was used with song as sole reward. In the experimental cages (70×30×45 cm) birds could peck at red response keys (diameter: 1 cm) with a red light-emitting diode (LED) in the center (diameter 2 mm) fitted into the rear wall. Pecking one key triggered one playback of the song assigned to it (tutor song or novel song). A custom-built control-registration unit kept a datalog and controlled the playbacks (soundchip Oki MSM6388, Tokyo, Japan). Songs were broadcast at 70 dB peak amplitude (CEL-231 sound-level meter) at 30 cm from the loudspeaker (Quart 250 or JBL Control 1), which was fitted behind an opening (diameter 9 cm) in the rear, halfway between the pecking keys. Songs played when either of the keys was pecked were interchanged daily so as to control for possible side preferences. The day after a bird started pecking both response keys, was designated day 1 of the preference test, which lasted 4 consecutive days. Most birds learned the task without training; those who had not started to peck the keys after 4 days were trained in sessions as described elsewhere [54]. The preference for the father’s song (i.e. the tutor song) was calculated by dividing the number of pecks for the father’s song by the grand total over the 4 days. Throughout the preference testing period, food and water were available ad libitum.

### Song (re-) Exposure

The birds were divided into three experimental groups. All birds received the same treatment throughout, except for the category of songs heard before being anesthetized and perfused. At 672±27 days after hatching, birds were placed in a cage in a sound-attenuating chamber for acclimatization and 2–3 days later, birds were either exposed to a recording of their tutor song (group TUTOR, N = 9), to their own song (group BOS, N = 11) or to a novel song of an adult zebra finch male (group NOVEL, N = 9). The subjects had never heard the novel songs before, and the songs were recorded from males that were unrelated to the tutor males. Novel songs used for the preference test were different from those used for playback before perfusion. Lights were switched off 15 minutes before onset of playbacks to keep movements that could induce IEG expression to a minimum and to prevent the birds from vocalizing. During 30 minutes, song segments of 6.1±0.7 s were played back at random intervals by a custom-made playback device (with soundchip Oki MSM6388, Tokyo, Japan) set to a rate of 60 random playbacks/hour. Songs were broadcast at 70 dB SPL peak amplitude at the point where the bird’s head was during the playback (CEL-231 sound-level meter). The birds remained in darkness for one hour after the end of song playback, when they were sacrificed. Continuous tape recording revealed that none of the birds sang while they were in darkness. Permission to perform this experiment was obtained from the Animal Experiments Committee of Leiden University (UDEC 00071).

### Song Recording and Analysis

Prior to the playback experiments, the undirected songs [56] of tutor, experimental and novel birds had been recorded with a Sennheiser MKH40 microphone (Sennheiser Electronic KG, Wedemark, Germany) and a Sony TC-D5 Pro II recorder (Sony Corporation, Japan). Recording of the bird’s own songs took place when the experimental birds were adult (over 120 days of age) in a sound-attenuating chamber. Songs were digitized using Signal–Rts software (Engineering Design, Belmont, MA, USA) with a sample rate of 20.5 kHz. For each experimental bird and each tutor, a sonogram was made of a representative motif using Avisoft SASLab Pro software (Berlin, Germany). Songs from the three categories (tutor songs, novel songs and the songs of the experimental males) were recorded under the same circumstances and their sonograms were produced in the same manner. Three independent observers, who did not know the origin of the sonograms, compared the sonograms of motifs of each of the experimental males in the three groups with that of the tutor motif. In addition, the observers were asked to compare 14 sonograms of tutor songs with sonograms of songs from males that were unrelated to the tutors and had always been in different rooms. Subsequently, for each experimental bird, the percentage of elements shared with the tutor song was calculated in relation to the number of different elements in the tutor song (cf. [25]). The percentage of elements that an unfamiliar bird shared with the tutor song was calculated in relation to the number of different elements in the tutor song to obtain a measure of the chance level of song element sharing. An element was defined as a single continuous vocalization separated from other vocalizations by either a short silent interval or by a major change in harmonic structure [57], [58]. Kendall’s coefficient of concordance for the three observers was 0.83. The mean scores of the three observers were used for analysis.

### Immunocytochemistry

One hour after the end of exposure to the stimulus song, birds received injections of 0.06 ml Nembutal (i.m.), and they were subsequently perfused with 0.2% heparin in saline and a fixative (4% paraformaldehyde in 0.1 M phosphate-buffered saline (PBS), pH 7.4). The brains were dissected out and placed in fixative for 3 hours, after which they were placed in a 30% sucrose solution overnight. Free floating sections (40 µm) were made using a freezing microtome, and placed in 0.1 M PBS (pH 7.4). The sections were then incubated in 3% H_2_O_2_ in PBS for 15 minutes, rinsed in PBS and incubated with normal goat serum for 30 minutes. Sections were rinsed in PBS again, and incubated at 4°C for 20 h with the primary antibody. We used polyclonal antibodies against egr-1 (C-19, sc-189), dilution 1∶1000, raised in rabbits (Santa Cruz Biotechnology, Santa Cruz, CA, USA). Specificity of this antiserum for activity-induced Zenk expression has been confirmed previously by Western blots of the zebra finch brain in which the antiserum recognized a single band of the 62.5-kDa protein [24]. Sections were then rinsed in PBS, incubated with goat-anti-rabbit for 1 hour and rinsed in PBS again. Staining involved incubation for 1 h in ABC (avidin/biotinylated enzyme complex; Vector Laboratories, Burlingame, CA, USA), rinsing in PBS, followed by rinsing in acetic acid and incubation in a diaminobenzidine medium for 25 min with 0.034% H_2_O_2_ added during the final 15 minutes. Sections were then rinsed in acetic acid and mounted onto slides using gelatin. Control sections were subjected to the same procedure, but were not incubated with the primary antibody. Alternate sections were stained for acetylcholinesterase to facilitate identification of brain structures.

### Image Analysis

The number of immunopositive cell nuclei in a standard size frame of 0.3×0.4 mm was counted in four sections in the HVC, RA and Area X. For all three structures, the counting frame was placed in the centre of the nucleus. For each brain region, mean values of the four sections were used to determine the number of immunoreactive cells. Digital photographs of the brain areas were made with a Nikon Coolpix 950 camera (Nikon Corporation, Tokyo, Japan) at 200x magnification. Image analysis was carried out with a PC-based system using KS400 version 3.0 software (Carl Zeiss Vision, Oberkochen, Germany). A program was developed in KS400 to quantify the number of immunoreactive cells in the brain areas of interest [59]. The immunoreactive cells and cell clusters, with an empirically determined optical density of 0.05 higher than the mean optical density of the background in the image, were selected automatically. Only those structures with a circular shape factor higher than 0.1 and an area greater than 10.0 µm^2^ were considered. The circular shape factor was defined as 4π×AREA)/perimeter^2^. Cell clusters were divided by a mean cell size that was empirically determined per brain region. The experimenter checked the selection made by the image analysis system and deselected artifacts manually. Counting was done ‘blind’ as to the experimental history of the subjects.

### Statistical Analyses

All data were natural log transformed before statistical analysis. A repeated measurements ANOVA with factors brain region (HVC, RA, Area X) as repeated measures and group (TUTOR, BOS, NOVEL) as between-subjects factor was used to investigate the effects of the different stimuli on the sampled brain regions. One-way ANOVA’s were performed on each brain region. Data were analyzed using SPSS 12.0.1.

## References

[1] Brainard MS, Doupe AJ (2000). Auditory feedback in learning and maintenance of vocal behaviour.. Nature Rev Neurosci.

[2] Brainard MS, Doupe AJ (2002). What songbirds teach us about learning.. Nature.

[3] Bolhuis JJ, Eda-Fujiwara H (2003). Bird brains and songs: neural mechanisms of birdsong perception and memory.. Anim Biol.

[4] Bolhuis JJ, Gahr M (2006). Neural mechanisms of birdsong memory.. Nature Rev Neurosci.

[5] Bolhuis JJ, Zeigler HP, Marler P (2008). Chasin’ the trace: The neural substrate of bird song memory..

[6] Mooney R (2009). Neural mechanisms for learned birdsong.. Learn Mem.

[7] Bolhuis JJ, Eda-Fujiwara H (2010). Birdsong and the brain: The syntax of memory.. NeuroReport.

[8] Doupe AJ, Kuhl PK (1999). Birdsong and human speech: common themes and mechanisms.. Annu Rev Neurosci.

[9] Bolhuis JJ, Okanoya K, Scharff C (2010). Twitter evolution: Converging mechanisms in birdsong and human speech.. Nature Rev Neurosci.

[10] Moorman S, Bolhuis JJ, Bolhuis JJ, Everaert, M (2012). Behavioral similarities between birdsong and spoken language..

[11] Konishi M (1985). Birdsong: from Behavior to Neuron.. Annu Rev Neurosci.

[12] Funabiki Y, Konishi M (2003). Long memory in song learning by zebra finches.. J Neurosci.

[13] Gobes SMH, Bolhuis JJ (2007). Bird song memory: A neural dissociation between song recognition and production.. Curr Biol.

[14] Gobes SMH, Fritz J, Bolhuis JJ (2012). Neural mechanisms of auditory learning and memory in songbirds and mammals In: Bolhuis JJ, Everaert, M, editors. Birdsong, Speech & Language. Cambridge, MA: MIT Press.. In press.

[15] Reiner A, Perkel DJ, Bruce LL, Butler AB, Csillag A (2004). Revised nomenclature for avian telencephalon and some related brainstem nuclei.. J Comp Neurol.

[16] Jarvis ED, Güntürkün O, Bruce L, Csillag A, Karten H (2005). Avian brains and a new understanding of vertebrate brain evolution.. Nature Rev Neurosci.

[17] Nottebohm F, Hauser M, Konishi M (2000). The anatomy and timing of vocal learning in birds..

[18] Doupe AJ, Perkel DJ, Reiner A, Stern EA (2005). Birdbrains could teach basal ganglia research a new song.. Trends Neurosci.

[19] Mello CV, Vicario DS, Clayton DF (1992). Song presentation induces gene expression in the songbird forebrain.. Proc Natl Acad Sci USA.

[20] Mello CV, Clayton DF (1994). Song-induced ZENK gene expression in auditory pathways of songbird brain and its relation to the song control system.. J Neurosci.

[21] Mello CV, Nottebohm F, Clayton DF (1995). Repeated exposure to one song leads to a rapid and persistent decline in an immediate early gene’s response to that song in zebra finch telencephalon.. J Neurosci.

[22] Chew S, Vicario D, Nottebohm F (1996). A large-capacity memory system that recognizes the calls and songs of individual birds.. Proc Natl Acad Sci USA.

[23] Jarvis E, Nottebohm F (1997). Motor-driven gene expression.. Proc Natl Acad Sci USA.

[24] Mello CV, Ribeiro S (1998). ZENK protein regulation by song in the brain of songbirds.. J Comp Neurol.

[25] Bolhuis JJ, Zijlstra GGO, den Boer-Visser AM, Van der Zee EA (2000). Localized neuronal activation in the zebra finch brain is related to the strength of song learning.. Proc Natl Acad Sci USA.

[26] Bolhuis JJ, Hetebrij E, den Boer-Visser AM, de Groot JH, Zijlstra GGO (2001). Localized immediate early gene expression related to the strength of song learning in socially reared zebra finches.. Eur J Neurosci.

[27] Terpstra NJ, Bolhuis JJ, den Boer-Visser AM (2004). An analysis of the neural representation of bird song memory.. J Neurosci.

[28] Phan ML, Pytte CL, Vicario DS (2006). Early auditory experience generates long-lasting memories that may subserve vocal learning in songbirds.. Proc Natl Acad Sci USA.

[29] London SE, Clayton DF (2008). Functional identification of sensory mechanisms required for developmental song learning.. Nat Neurosci.

[30] Gobes SMH, Zandbergen MA, Bolhuis JJ (2010). Memory in the making: Localized brain activation related to song learning in young songbirds.. Proc Roy Soc Lond B.

[31] Solis MM, Brainard MS, Hessler NA, Doupe AJ (2000). Song selectivity and sensorimotor signals in vocal learning and production.. Proc Natl Acad Sci USA.

[32] Margoliash D, Konishi M (1985). Auditory representation of autogenous song in the song system of white-crowned sparrows.. Proc Natl Acad Sci USA.

[33] Margoliash D (1986). Preference for autogenous song by auditory neurons in a song system nucleus of the white-crowned sparrow.. J Neurosci.

[34] Nottebohm F (1981). A brain for all seasons: Cyclical anatomical changes in song control nuclei of the canary brain.. Science.

[35] Mello CV, Jarvis E, Zeigler HP, Marler P (2008). Behavior-dependent expression of inducible genes iin vocal learning birds..

[36] Prather JF, Mooney R, Zeigler HP, Marler P (2008). Song-selective neurons in the songbird brain: synaptic mechanisms and functional roles..

[37] Theunissen FE, Amin N, Shaevitz S, Woolley SMN, Fremouw T, Zeigler HP, Marler P (2008). Song selectivity and the songbird brain..

[38] Prather JF, Peters S, Nowicki S, Mooney R (2008). Precise auditory-vocal mirroring in neurons for learned vocal communication.. Nature.

[39] Prather JF, Nowicki S, Anderson RC, Peters S, Mooney R (2009). Neural correlates of categorical perception in learned vocal communication.. Nat Neurosci.

[40] Nick TA, Konishi M (2005). Neural song preference during vocal learning in the zebra finch depends on age and state.. J Neurobiol.

[41] Solis MM, Doupe AJ (2000). Compromised neural selectivity for song in birds with impaired sensorimotor learning.. Neuron.

[42] Aamodt SM, Nordeen EJ, Nordeen KW (1995). Early isolation from conspecific song does not affect the normal developmental decline of NMDA receptor binding in an avian song nucleus.. J Neurobiol.

[43] Heinrich JE, Singh TD, Nordeen KW, Nordeen EJ (2003). NR2B downregulation in a forebrain region required for avian vocal learning is not sufficient to close the sensitive period for song learning.. Neurobiol Learn Mem.

[44] Livingston FS, White SA, Mooney R (2000). Slow NMDA-EPSCs at synapses critical for song development are not required for song learning in zebra finches.. Nat Neurosci.

[45] Nordeen KW, Nordeen EJ (2004). Synaptic and molecular mechanisms regulating plasticity during early learning.. Ann NY Acad Sci.

[46] Aamodt SM, Nordeen EJ, Nordeen KW (1996). Blockade of NMDA receptors during song model exposure impairs song development in juvenile zebra finches.. Neurobiol Learn Mem.

[47] Basham ME, Nordeen EJ, Nordeen KW (1996). Blockade of NMDA receptors in the anterior forebrain impairs sensory acquisition in the zebra finch (*Poephila guttata*) *Neurobiol*.. Learn Mem.

[48] Velho TAF, Pinaud R, Rodrigues PV, Mello CV (2005). Co-induction of activity-dependent genes in songbirds.. Eur J Neurosci.

[49] Gobes SMH, Ölveczky BP (2011). The sensorimotor nucleus NIf is required for normal acquisition and production of birdsong. *Program No. 303.15. 2011 Neuroscience Meeting Planner*. Washington, DC: Society for Neuroscience.. Online.

[50] Volman SF (1993). Development of neural selectivity for birdsong during vocal learning.. J Neurosci.

[51] Cardin JA, Raksin JN, Schmidt MF (2005). Sensorimotor nucleus nif is necessary for auditory processing but not vocal motor output in the avian song system.. J Neurophysiol.

[52] Coleman MJ, Mooney R (2004). Synaptic transformations underlying highly selective auditory representations of learned birdsong.. J Neurosci.

[53] Riebel K, Smallegange IM, Terpstra NJ, Bolhuis JJ (2002). Sexual equality in zebra finch song preference: evidence for a dissociation between song recognition and production learning.. Proc R Soc Lond B.

[54] Houx B, ten Cate C (1999). Song learning from playback in zebra finches: is there an effect of operant contingency?. Anim Behav.

[55] Riebel K (2000). Early exposure leads to repeatable preferences for male song in female zebra finches.. Proc R Soc Lond B.

[56] Zann R (1996). The zebra finch: a synthesis of field and laboratory studies.. Oxford: Oxford UP.

[57] Sturdy CB, Phillmore LS, Weisman RG (1999). Note types, harmonic structure, and note order in the songs of zebra finches (*Taeniopygia guttata*).. J Comp Psychol.

[58] Sturdy CB, Phillmore LS, Price JL, Weisman RG (1999). Song-note discriminations in zebra finches (*Taeniopygia guttata*): categories and pseudocategories.. J Comp Psychol.

[59] Gobes SMH, ter Haar SM, Vignal C, Vergne AL, Mathevon N (2009). Differential responsiveness in brain and behavior to sexually dimorphic long calls in male and female zebra finches.. J Comp Neurol.

[60] Moorman S, Mello CV, Bolhuis JJ (2011). From songs to synapses: Molecular mechanisms of birdsong memory.. BioEssays.

